# Microbial reductions and physical characterization of chitosan flocs when using chitosan acetate as a cloth filter aid in water treatment

**DOI:** 10.1371/journal.pone.0262341

**Published:** 2022-01-21

**Authors:** Hemali H. Oza, Eleanor B. Holmes, Emily S. Bailey, Collin K. Coleman, Mark D. Sobsey

**Affiliations:** 1 Gangarosa Department of Environmental Health, Rollins School of Public Health, Emory University, Atlanta, GA, United States of America; 2 Department of Environmental Sciences and Engineering, Gillings School of Global Public Health, University of North Carolina, Chapel Hill, NC, United States of America; 3 Texas Tech University Health Sciences Center, Graduate School of Biomedical Sciences, Julia Jones Matthews Department of Public Health, Abilene, TX, United States of America; Purdue University, UNITED STATES

## Abstract

The World Health Organization (WHO) estimates 2.1 billion people lack access to safely managed water. Cloth filtration is often employed in rural and developing communities of South Asia for point-of-use water treatment, but bacteria and viruses are too small for efficient removal by this filtration method. Chitosan is a biodegradable, cationic, organic polymer derived from the chemical treatment of chitin that acts as a coagulant and flocculant of contaminant of microbes and other particles in water, thereby facilitating filtration of microbes. This research 1) evaluated the use of chitosan acetate as a pre-treatment coagulation-flocculation process followed by cloth filtration for microbial reductions and 2) assessed floc particle size under three stirring conditions. *E*. *coli* KO11 bacteria and MS2 coliphage virus removals were quantified using culture-based methods. Chitosan acetate coagulation-flocculation pre-treatment of water, followed by cloth filtration, met or exceeded the protective (2-star) WHO performance levels for bacteria (2 log_10_ reduction) and viruses (3 log_10_ reduction), and filtrate turbidity was consistently reduced to < 1 NTU, meeting United States Environmental Protection Agency (EPA) and WHO targets.

## Introduction

Approximately 884 million people lack access to improved drinking water, of which 90% are located in rural and low-income settings [1–3]. This puts developing and rural people and communities at greater risk of being exposed to waterborne pathogens and their resulting diseases by putting the responsibility of acquiring, treating, and safely storing water on many of the poorest and lowest-resource households and communities [4, 5]. As utilities and governments work to improve implementation and management of conventional water treatment and delivery infrastructure in the coming years, there is still remains a current need for timely, achievable, and economical strategies and technologies for safe water provision.

In response to this service gap, point-of-use (POU) technologies, also known as household water treatment (HWT) technologies, that reduce contaminants are being widely promoted and used in many rural settings and low-to-middle-income countries (LMICs). Applied by individuals prior to consumption, these technologies are designed to treat water and/or prevent further contamination of stored water at the household level [6]. Popular POU treatment technologies include ceramic water filters (CWF), BioSand (intermittent flow slow sand) filters, cloth filters, solar disinfection, free chlorine disinfection, and coagulation.

To evaluate and provide guidance on the performance of these various technologies, WHO created HWT performance level targets based on Log_10_ reduction values (LRVs) of bacteria, viruses, and protozoa to achieve specified health outcomes based on epidemiological evidence. These targets encompass three levels: limited protection, protective, and highly protective [7]. Single-barrier POU technologies, which rely on only one microbial reduction process (e.g., filtration for removal or chemical disinfection for deactivation, etc.), do not consistently achieve the higher performance targets. Hence, there is a need for a simple and practical multi-barrier technology approach that improves the efficacy of drinking water treatment and increases pathogen removal and/or deactivation [8].

The coagulation-flocculation-sedimentation process is a water treatment technology using chemical coagulants that are usually positively charged in water and bind with negatively charged particles such as clay, organic matter, and microorganisms. Binding of charged particles creates flocs of increasing size that can then settle out under the influence of gravity or be removed by filtration [9]. Coagulation-flocculation and sedimentation procedures are crucial in reducing turbidity levels of organic and inorganic particulate matter as well as pathogens. While turbidity is not a direct health risk indicator, studies have suggested a strong relationship between turbidity reduction and protozoan removal [10]. Furthermore, WHO recommends reducing turbidity to less than one Nephelometric Turbidity Unit (NTU) for health protection [11]. Combining coagulation-flocculation and filtration as a multi-barrier technology has the potential to be more effective at removing pathogenic contaminants than either process alone [12].

Chitosan is a biodegradable, linear polysaccharide of repeating N-acetyl-D-glucosamine and D-glucosamine monomers that is derived from the chemical treatment of chitin. When added to water, chitosan acts as a coagulant to facilitate the physical removal, by both sedimentation and filtration, of captured viruses, bacteria, protozoans and other colloidal particulate matter as large flocs that can be settled or filtered out [12]. Chitosan is non-toxic and has been used in a variety of foods, nutritional supplements, cosmetic products, drug therapies, and other medical applications [13]. In contrast to inorganic coagulants, chitosan is able to achieve microbial reductions without the need for precise control of pH or coagulant dose, making it a practical candidate for use in water treatment as a POU pre-treatment for filtration [12].

Simple saree or cloth filtration is a type of POU filtration method that is popular among rural and low-income, South Asian communities. Saree cloth is a type of garment typically worn by South Asian women and is comprised of tightly woven threads, commonly made of cotton or silk. Due to the tightly woven threads, some contaminants are prevented from passing through, making the cloth a potential POU technology if properly optimized [14]. Cloth filtration has not been studied extensively for microbial reductions from drinking water except for *Vibrio cholerae* bacteria that are associated with comparatively larger copepods in water. Because the size of contaminant particles is known to be a key factor influencing LRVs by filtration processes, the large pore sizes of cloth filters compared to other POU filtration technologies makes it inefficient in retaining viruses, bacteria and protozoan pathogens that are not associated with larger particles such as copepods [14, 15]. The attraction of using cloth filtration stems from its household availability as scraps of available cloth for ease of use to filter water at the household level.

A chitosan coagulation pre-treatment step prior to filtration has the potential to greatly improve microbial and turbidity reductions by trapping large floc particles [12]. However, better understanding of chitosan floc formation and particle size is crucial to maximizing its ability to function as an effective coagulant of viruses and bacteria, especially when used in conjunction with cloth filtration, where the size of particles being filtered is critical for maximizing LRVs. These aspects of chitosan coagulation-flocculation and filtration are the basis of this lab study.

The goal of this study was to quantify *Escherichia coli* KO11 bacteria, MS2 male-specific (F+) coliphage viruses, and turbidity reductions in seeded natural test water of defined quality when subjected to chitosan coagulation pre-treatment under different coagulation-flocculation stirring conditions, followed by sedimentation and cloth filtration. Reductions of bacteria and viruses were compared to the three levels of WHO HWT performance targets. An additional goal was to assess the effects of water quality and flocculation stirring conditions on floc size formation and distribution using a particle size analyzer (PSA) to characterize and optimize floc size for cloth filtration and pathogen reduction.

## Materials and methods

### Chitosan

Chitosan acetate (Food Grade Chitosan purchased from *Sarchem Laboratories*, *Inc*. in powder form) was chosen for this study based on the results of previous studies comparing chitosan hydrochloride, chitosan acetate, and chitosan lactate. In that study, the use of chitosan acetate resulted in higher LRVs, ranging from 2.8–4.5 Log_10_-reduction of MS2 *coliphage*, compared to the other acid-modified chitosans tested [12]. The degree of deacetylation of the chitosan acetate was 90.3% and the pH in reagent water was 4.2.

### Cloth filters

A 100% cotton linen material (200 thread count; determined by microscopy in this study to have a single layer a pore size of 100 μM which is illustrated in S1 Fig in S1 File), was used for cloth filters and 12 layers of the square material were stacked to create a porous filter medium. A 1% solution of commercial bleach was made in deionized water from a concentrated commercial bleach of 87,000 mg/L available sodium hypochlorite to produce an 870 mg/L solution of available sodium hypochlorite. The cloth filters were soaked in this 1% bleach solution for 30 minutes, rinsed with deionized water, and air dried for at least 24 hours. The cloth filters were slightly dampened in deionized water and secured to a polyvinyl chloride (PVC) column of 15.2 cm diameter and 7.6 cm in length by tightly wrapping rubber bands around the column. The filter-column apparatus was placed over 2 L beakers used to collect the treated water filtrate.

### Water source

Two types of challenge waters were used for both microbial and PSA experiments: a natural surface lake water, and the same source of natural surface lake water supplemented with 10 ml/L of pasteurized sewage to create a water with higher organic content as might be found in low resource settings. The lake water was collected from University Lake in Chapel Hill, N.C., U.S.A., and primary effluent sewage, which was pasteurized and added to lake water as the second challenge water type, was collected from the Orange Water and Sewer Authority (OWASA) Mason Farm Wastewater Treatment Plant, Chapel Hill, N.C., U.S.A. To pasteurize, the sewage sample was heated to 70°C for 30 minutes. Water samples used for both challenge waters for microbial experiments and for non-sewage amended PSA experiments were collected on August 18, 2018, and water samples used for sewage amended PSA experiments were collected on March 1, 2019; sampled water was stored at 4°C. Water quality parameters for these waters can be found in S1 Table in S1 File.

### Test microbes

*Escherichia coli* KO11 (ATCC #55124) was used as an indicator for pathogenic enteric bacteria. MS2 male-specific (F+) coliphage (bacteriophage) (ATCC #15597-B1) was used as the model virus because its molecular characteristics, composition, and physical properties are similar to those of other human enteric viruses of health concern, such as noroviruses, enteroviruses, astroviruses and hepatitis A and E viruses. Removal of MS2 coliphage is expected to be similar to removal of other human enteric viruses [8]. Stocks of microbes were propagated in the laboratory and dosed into challenge waters to achieve an initial concentration ≥ 10^6^ organisms per 100 mL to provide a microbial concentration sufficient to measure as much as a 6 Log_10_ reduction.

### Experimental design

Prior to treatment with chitosan, a sample of influent water (containing added test microbes and also pasteurized sewage for certain experiments) was taken for analysis. Two liters of influent challenge waters, specifically lake water, with added *E*. *coli* KO11 and MS2 coliphage particles and in half of experiments, also amended with pasteurized sewage, were prepared for each filter and placed in separate containers. Chitosan was dissolved in water at 2 g/L as a concentrated stock and a 10 mg/L dose was added as a small volume to each 2 L of challenge water. Control waters for these experiments received 0 mg/L of chitosan (S2 Table in S1 File). The dosed water was then mixed using a jar-test-flocculator apparatus containing paddle blade stirrers to initiate the coagulation-flocculation processes according to the conditions detailed in S3 Table in S1 File.

After sedimentation but prior to filtration, a sample from the middle of the flocculation jar and one inch below the surface was taken for analysis. The remaining supernatant water was passed through the cloth filter apparatus by gravity flow, and effluent water samples were taken for analysis. Samples were analyzed to determine microbial concentrations, turbidity, and pH levels. The difference between Log_10_ influent and effluent concentrations of *E*. *coli* KO11 and coliphage MS2 were used to calculate LRVs for microbes and turbidity levels. LRVs were calculated from differences in Log_10_ concentrations at various sampling points to assess removal due to each of the following processes: filtration alone, coagulation-flocculation-sedimentation pre-treatment alone, isolated effects of filtration alone after pre-treatment, and both pre-treatment and filtration together. Experiments were run in triplicate.

### Microbiological methods

Bacteria detection and enumeration: Bacterial stocks were prepared as described in Abebe et al., 2016 and stored at -80°C [12]. In brief, a ~200 μL scrape of frozen *E*. *coli* KO11 stock culture of 10^9^ colony forming units (CFU)/1 mL was dispensed into a flask of 50 mL of tryptic soy broth (TSB) (Difco) with 34 μg/mL of chloramphenicol antibiotic, and incubated on a shaker at 100 rpm for 18–24 hours at 37°C. The culture was then centrifuged at 3000x gravity for 10 minutes at 4°C, and the sedimented cells were washed three times in succession with phosphate buffer. Approximately 20 mL of the washed *E*. *coli* KO11 suspension were added per 10 liters of challenge water, producing a concentration of ~10^6^ CFU/mL of *E*. *coli* KO11.

*E*. *coli* KO11 was enumerated as colony forming units (CFU) using 100 x 15 mm Petri plates of Tryptic Soy Agar (TSA) (Difco) supplemented with 30 mg/L neutral red, 10 g/L lactose, and 34 μg/mL of chloramphenicol antibiotic at 12–15 mL/plate as described in Standard Methods part 9215 C [16]. Serial 10-fold dilutions were made for all water samples, and 100 μL of sample were spread on duplicate plates. Plates were inverted, incubated for 18 to 24 hours at 37°C, and the resulting characteristic *E*. *coli* colonies were counted.

Virus propagation and enumeration: Stock of *E coli* F_amp_ host bacteria was prepared as described in Abebe et al., 2016 and stored at -80°C [12]. A 1 mL frozen stock sample of MS2 coliphage, with a titer of 1x10^11^ plaque forming units (PFU) /mL, was thawed, diluted and added to challenge waters to produce 10^6^−10^8^ PFU/mL MS2 suspension. Double agar layer (DAL) plaque assays for MS2 enumeration in water samples were used as described in US EPA Method 1601 [17]. Log-phase *E coli* host was prepared the day of the experiment using optical density to confirm adequate growth. On the day of experimental runs, 0.7x TSA (28 g/L) was supplemented with 0.05 M, 15 μg/mL, 15 μg/mL of MgCl_2_, streptomycin, and ampicillin, respectively, and tempered to 55–65°C. Water samples of 100 μL, 250 μL of the log phase *E*. *coli* F_amp_ broth culture, and 15 μg/mL streptomycin and ampicillin were added per 5 mL of molten 0.7x TSA in 10 mL glass tubes that were flamed, then swirled by hand, and poured over the bottom 1.5x TSA layer. Samples were plated in duplicate. Plates were inverted and incubated for 18 to 24 hours at 37°C, and the resulting plaques were counted to determine MS2 concentrations expressed as PFU per mL.

### Turbidity and pH

For all samples, turbidity was measured with a turbidimeter (Hach 2100AN Turbidimeter, Hach, Loveland CO) and pH was measured with a pH meter (pH Meter Model 215 Denver Instrument Company) and a combination electrode.

### Floc size formation and size distribution

The particle size analyzer, Mastersizer 3000 by Malvern, was used to analyze floc formation and size characterization resulting from coagulation-flocculation by the addition of 10 mg/L of chitosan acetate to the two challenge waters (natural lake water and natural lake water amended with 1% pasteurized sewage in lake water by volume) (S4 Table in S1 File). The settings, as detailed in S5 Table in S1 File, were set prior to running the water sample.

After settings were established, the Mastersizer was initialized, background measurements were taken, and sample measurements were taken. The mixing procedures that were used for microbial assessments were also used in these experiments, as detailed in S4 Table in S1 File. Non-pasteurized-sewage amended raw challenge water with 10 mg/L added chitosan acetate was passed through the Mastersizer. The 50^th^ percentile floc size was measured and recorded over the entire coagulation-flocculation and sedimentation process. After sampling was complete, soapy water and then deionized water was run through the tubing and the Mastersizer to remove any residual flocs or natural lake water. Deionized water was passed through the tubing, and tube endings were submerged in deionized water to prevent the Mastersizer measurement cell from drying out. All experiments were run in triplicate.

### Statistical analysis

Mean Log_10_ reductions of *E*. coli and MS2 were calculated from replicate LRVs per sample type. A linear regression analysis was conducted among *E*. *coli* KO11, MS2 coliphage, and turbidity reductions by pooling data points by the parameters of challenge water type, chitosan dose, stirring condition, and sampling point. Estimate mean difference, standard error, t-values, p-values, and 95% confidence intervals of Log_10_ reductions were reported. The last 30 data points per PSA experiment were pooled by stirring condition and challenge water into sets of *n = 90*. A t-test for parametric datasets was used to test for significant differences in particle size among challenge waters and stirring conditions. Statistical analyses were done using R Statistical Software.

## Results

**Fig 1:**
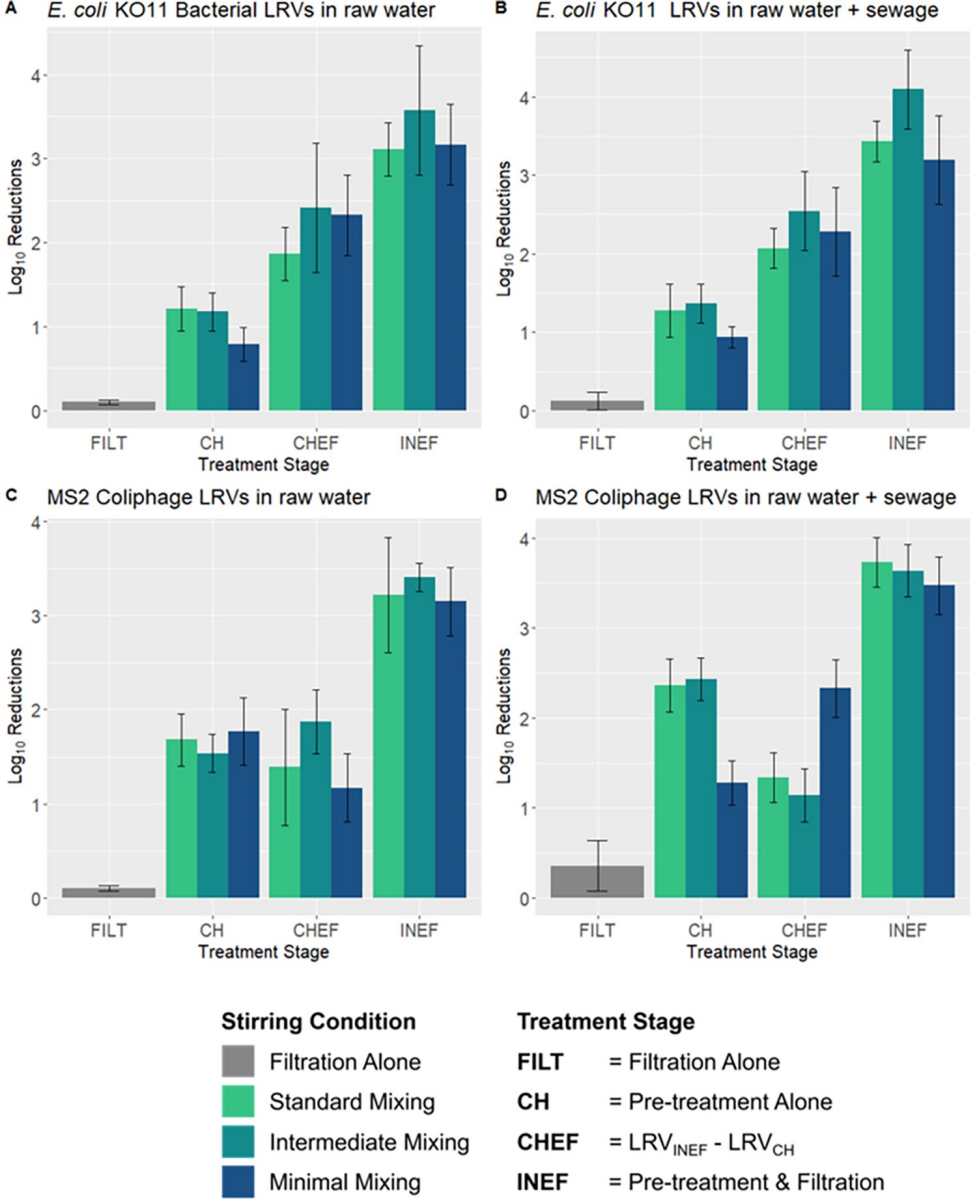
Mean LRVs and their 95% confidence limits (as bar whiskers) by test microbe, test water, stirring condition, and sampling point. (A) *E*. *coli* KO11 bacterial LRVs in raw water; (B) *E*. *coli* KO11 bacterial LRVs in raw water + 1% pasteurized sewage; (C) MS2 bacteriophage LRVs in raw water; (D) MS2 bacteriophage LRVs in raw water + 1% pasteurized sewage.

Chitosan pre-treatment by coagulation-flocculation with a 10 mg/L dose of chitosan followed by cloth filtration together resulted in average LRVs of > 3 for both *E coli* and MS2 in both waters (Fig 1 and S7 Table in S1 File), for all three stirring conditions. Effluent turbidity after pre-treatment and filtration was < 1 NTU (S6 Table in S1 File).

### *E*. *coli* KO11 and MS2 coliphage LRVs

Note that the variable CHEF represents the LRV due to the isolated effects of filtration (after chitosan pre-treatment). This variable was calculated by subtracting the LRVs achieved by pre-treatment alone from the LRVs achieved by pre-treatment and filtration together.

When solely employing filtration (no chitosan pre-treatment), 0.10 (± 0.03) Log_10_ reductions were achieved for *E*. *coli* KO11 in non-sewage amended samples. Under standard and intermediate stirring conditions, 1.2 (± 0.3) and 1.2 (± 0.2) Log_10_ reductions, respectively, were achieved solely due to chitosan pre-treatment. Intermediate stirring conditions achieved 3.6 (± 0.8) Log_10_ reductions due to the combined effects of pre-treatment and filtration, which was the highest among the three stirring conditions tested. Filtration alone resulted in 0.10 (± 0.1) Log_10_ reductions of *E*. *coli* KO11 in sewage amended samples. Intermediate stirring conditions resulted in the largest Log_10_ reductions of *E*. *coli* KO11 in sewage amended samples due to chitosan pre-treatment alone (1.4 ± 0.2) and pre-treatment combined with filtration (4.1 ± 0.5) (Fig 1 and S7 Table in S1 File).

When controlling for all other parameters, *E*. *coli* KO11 LRVs in sewage amended samples were on average 0.14 Log_10_ greater than resulting LRVs in non-sewage amended samples (*p-value = 0*.*094)* (Table 1). Intermediate stirring conditions, on average, resulted in higher LRVs than standard and minimal stirring conditions (Table 1). For the test microbe *E*. *coli* KO11, chitosan pre-treatment alone resulted in a significant estimated LRV difference of 1.01 Log_10_ reductions greater than filtration alone (*p-value < 1*.*0 × 10*^*−5*^), and pre-treatment followed by filtration resulted in a significant estimated LRV difference of 3.32 Log_10_ reductions greater than filtration alone (*p-value < 1*.*0 × 10*^*−5*^) (Table 1).

**Table 1:** Comparisons of estimated mean differences in average LRVs of *E*. *coli* KO11 and MS2 coliphage after controlling for selected parameters in linear regressions^1^.

	Comparison Parameters			Est. LRV Mean Diff.	Std. Error	Pr(>|t|)
	Parameter_A_ (LRV_A_) > Parameter_B_ (LRV_B_)					
***E*. *coli* KO11**	Stirring Conditions					
		Intermediate (2.5)	Minimal (2.1)	0.31	0.10	0.0024**
		Intermediate (2.5)	Standard (2.2)	0.28	0.10	0.0064**
		Standard (2.2)	Minimal (2.1)	0.03	0.10	0.74
	Treatment Stage					
		Pre-treatment Alone (1.1)	Filtration Alone (0.1)	1.01	0.11	< 1.0 × 10^−5^ ***
		Pre-treatment & Filtration (3.4)	Filtration Alone (0.1)	3.32	0.11	< 1.0 × 10^−5^ ***
**MS2 coliphage**	Stirring Conditions					
		Intermediate (2.3)	Minimal (2.2)	0.11	0.11	0.34
		Intermediate (2.3)	Standard (2.3)	0.04	0.11	0.73
		Standard (2.3)	Minimal (2.2)	0.07	0.11	0.54
	Treatment Stage					
		Pre-treatment Alone (1.8)	Filtration Alone (0.2)	1.61	0.13	< 1.0 × 10^−5^ ***
		Pre-treatment & Filtration (3.4)	Filtration Alone (0.2)	3.21	0.13	< 1.0 × 10^−5^ ***

^1^Using linear regressions, the estimated LRV mean difference was calculated for two parameters—stirring conditions and treatment stages. First, after controlling for water sample type (i.e., sewage and non-sewage amended samples) and treatment stage, estimated LRV mean differences among stirring conditions were calculated for *E*. *coli* KO11 and MS2 coliphage, separately. Second, after controlling for water sample type and stirring conditions, estimated LRV mean differences among treatment stages (i.e., Pre-treatment Alone and Pre-treatment & Filtration vs. Filtration Alone) were calculated for *E*. *coli* KO11 and MS2 coliphage, separately. Additional linear regression findings can be found in the supporting information under S8 Table in S1 File. LRVs listed next to the parameter were calculated by pooling all LRV results associated with the parameter by microbe type, *E*. *coli* KO11 and MS2; LRVs as averages are by parameter and were calculated separately.

** α = 0.010; 99% confidence-level.

*** α < 1.0 × 10^−5^; > 99.999% confidence-level.

Filtration alone achieved 0.10 (± 0.03) Log_10_ reductions of MS2 in non-sewage amended samples. Standard stirring conditions achieved the highest LRV due to chitosan pre-treatment alone (1.7 ± 0.3), and intermediate stirring conditions achieved the highest LRV due to pre-treatment and filtration, together (3.4 ± 0.2). Filtration alone resulted in 0.4 (± 0.3) Log_10_ reductions of MS2 in sewage amended samples. Under standard and intermediate stirring conditions, 2.4 (± 0.3) and 2.4 (± 0.2) Log_10_ reductions were achieved solely due to chitosan pre-treatment alone, respectively. Pre-treatment using standard stirring conditions combined with filtration resulted in the highest LRV for MS2 in sewage amended samples (3.7 ± 0.3) (Fig 1 and S7 Table in S1 File).

When controlling for all other parameters, average MS2 coliphage LRV differences for 1% pasteurized sewage-amended samples were found to be 0.27 Log_10_ higher than for non-pasteurized-sewage amended samples, which was significant at a 99% confidence level (*p-value = 0*.*0040)*. There was no significant difference of MS2 coliphage reductions when comparing the three stirring conditions (Table 1). Reductions due to chitosan pre-treatment alone were, on average, 1.61 Log_10_ higher than filtration alone (*p-value < 1*.*0 × 10*^*−5*^) (Table 1). Reductions due to the combined effects of chitosan pre-treatment and filtration were, on average, 3.21 Log_10_ reduction differences higher than filtration alone (*p-value < 0*.*00001*) (Table 1).

### Turbidity LRVs

Average turbidity LRV differences from intermediate stirring conditions and filtration had a mean difference of 0.24 Log_10_ reductions above the combined efforts of minimal stirring conditions during pre-treatment and filtration (*p-value = 0*.*025)*. Average Log_10_ turbidity reduction differences due to pre-treatment with intermediate, standard, and minimal coagulation-flocculation stirring conditions and filtration were all found to be significantly higher than Log_10_ turbidity reduction differences achieved by filtration alone. On average, intermediate, standard, and minimal stirring conditions for pre-treatment followed by filtration resulted in LRV reduction differences greater than those achieved by filtration alone, with LRV differences of 0.98 (p-value < 1.0 × 10^−5^), 0.84 (p-value < 1.0 × 10^−5^), and 0.73 (p-value < 1.0 × 10–5), respectively (S8 Table in S1 File).

### Filter pore size analysis

The cloth material used for the filters was observed under a light microscope to measure the approximate pore size (~100 μm) of a single cloth filter layer. The fibers of the used cloth were visibly agitated and frayed (box F), as compared to the new material (box E) (S2 Fig in S1 File).

### Floc size measurement analysis

**Fig 2:**
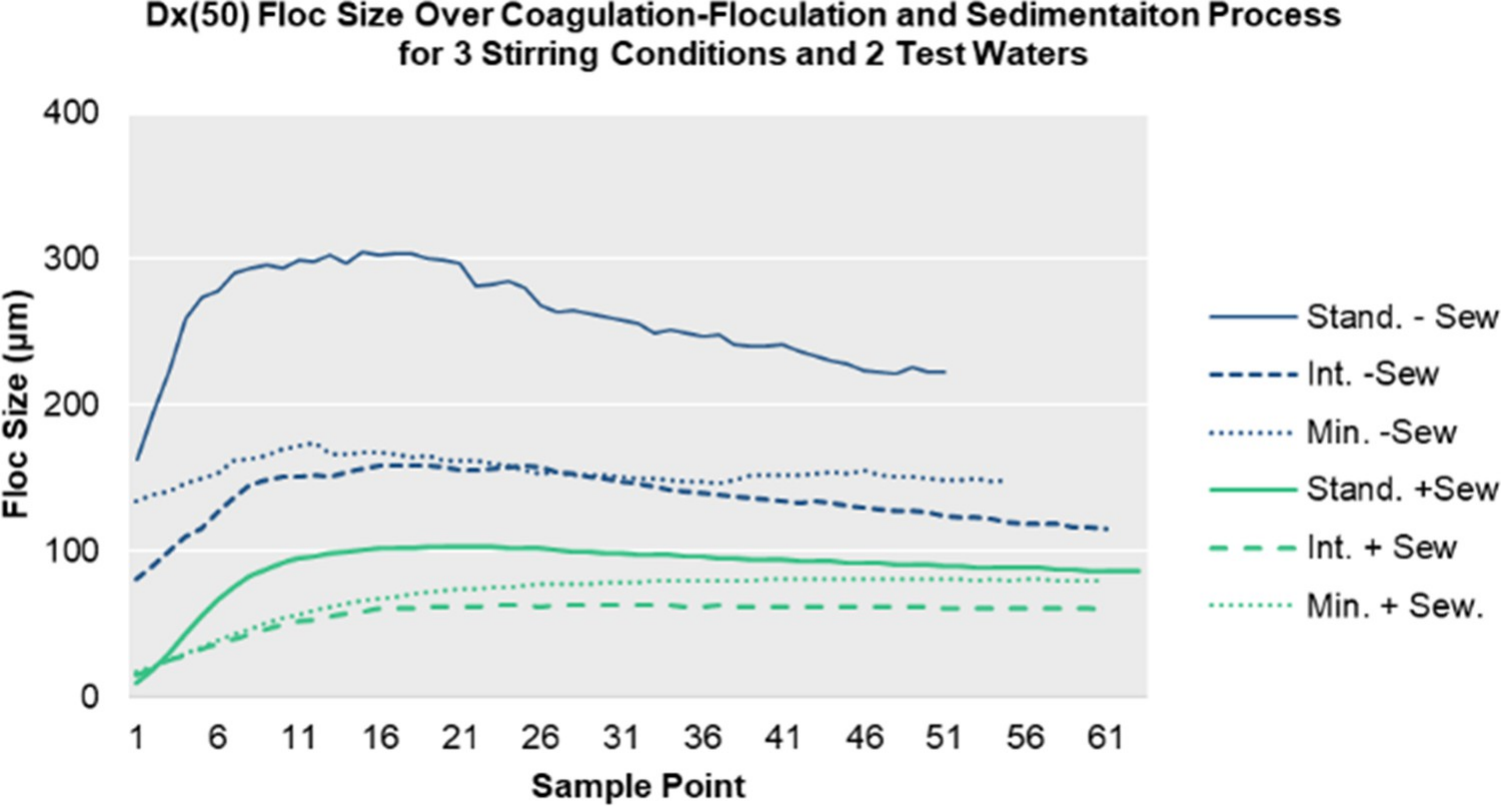
Average median floc size measurements for three stirring conditions and two challenge water types over the coagulation-flocculation sedimentation process.

As shown in Fig 2, the standard stirring conditions resulted the largest average floc sizes for both challenge waters, reaching 300 μM in standard water and 100 μM in standard water with sewage. The intermediate and minimum stirring conditions produced smaller average floc sizes for both challenge waters that were <100 μM (Fig 2 and S9 Table in S1 File). The non-sewage amended water samples from the August collection had significantly higher floc sizes than the floc sizes from sewage-amended water samples from the March collection (p < 0.000010) (S10 Table in S1 File). Standard stirring procedures were found to produce flocs significantly larger than those produced from intermediate and minimal procedures for both challenge water types (p < 0.000010) (S10 Table in S1 File). Intermediate stirring conditions produced significantly smaller flocs than standard and minimal stirring conditions for both challenge waters (p < 0.000010) (S10 Table in S1 File).

## Discussion

In this study, the effectiveness of three different coagulation-flocculation stirring conditions in two test surface waters at a 10 mg/L chitosan acetate dose as coagulation-flocculation pre-treatment followed by cloth filtration was evaluated for improving drinking water quality. Microbial reductions by chitosan coagulation-flocculation-sedimentation pre-treatment and cloth filtration were more than 3 Log_10_ greater than those by filtration alone (p-value < 0.00001) (Table 1). The dual barrier technology optimized in this study achieved the WHO “protective” performance requirements for viruses (3 log_10_) and bacteria (2 log_10_) (when using water collected in August 2018). Furthermore, turbidities of effluent water after pre-treatment and filtration using intermediate and standard stirring conditions in both challenge water types were consistently < 1 NTU, which meets turbidity guidance values of WHO and standards of US EPA [18].

Floc sizes formed after treatment with a 10 mg/L dose of chitosan acetate in non-sewage amended raw surface waters sampled in August were significantly larger than flocs formed by a 10 mg/L dose of chitosan acetate in raw water sampled from March and amended with 1% pasteurized sewage (S9 Table in S1 File). The reasons for these observed floc size differences were not specifically investigated in this study but may be due to chemical differences in water quality.

In both laboratory [19] and field studies [14] in Bangladesh saree cloth filtration achieved 2 Log_10_ reduction in *V*. *cholerae* and a 48% decrease in hospital cases of cholera. Because the combined use of chitosan and cloth filtration, met the WHO “protective” performance target, it is possible that adverse health effects also would be reduced, but field studies would be needed to document such beneficial health effects.

Colwell et al., 2002 also found that used or agitated saree cloth material had a smaller effective pore size than new saree cloth, with a single layer of used saree cloth having a pore size of 100–150 μm, and saree cloth folded four to eight times having a 20 μm pore size [14]. Similarly, a single later of the cloth filters in this current study had a pore size of ~100 μm. Cloth filters of the current study were washed and reused between experiments, but were not deliberately agitated to produce a smaller effective pore size. Had the cloth of this current study been agitated further to promote fraying and other physical alteration, LRVs may have been even greater than values observed. Because LRVs with repeated cloth usage over time were not evaluated specifically as an experimental variable, the impacts of repeated cloth usage on microbial reduction performance were not determined.

The results of the current study are generally consistent with those of previous studies that evaluated the performance of other natural organic polymers. Laboratory studies of *Moringa oleifera*, a plant-based coagulant, achieved 1–2 Log_10_ bacterial reductions [20, 21]. *S*. *potatorum*, another plant-based coagulant, and *M*. *oleifera*, when used in conjunction with filtration, achieved reductions of ~2-Log_10_ for bacteria and ~3-Log_10_ for viruses. However, while *M*. *oleifera* gave removals of seeded *E*. *coli* in challenge waters in a laboratory setting, it did not effectively remove coliform bacteria from stored village water [22]. Furthermore, other studies have documented secondary bacterial re-growth 24 hours after the initial *M*. *oleifera* treatment [20, 23]. Whether chitosan-pretreated and cloth filtered water causes bacteria regrowth was not investigated in this study and to our knowledge has not been studied by others. Additionally, it should be noted the aforementioned specific plant species used as coagulants are not always abundantly and readily available in some geographic regions [24], while chitosan is abundantly available worldwide.

Although the effectiveness of cloth filtration in removing bacteria spores, protozoans, or other microorganisms such as mycotic agents was not evaluated in this study, the relevant literature suggests cloth filtration may be able to remove such larger pathogens through size exclusion. Paper, nylon, and polyester filters are often recommended for removal of schistosomes and the Cyclops vector of guinea worm [25], and studies document their effectiveness at community and household levels [26]. While recommended for larger organisms or bacteria that attach to or associate with larger organisms, cloth filtration alone is not recommended for the removal of bacteria and viruses, and therefore, a multi-barrier approach for POUs employing cloth is required, such as with the addition of chitosan coagulation-flocculation pre-treatment as used in this study.

Average MS2 LRVs due to chitosan acetate coagulation-flocculation pre-treatment alone were, on average, 0.72 higher than *E*. *coli* KO11 LRVs, which was a significant finding (p-value < 0.000010). One possible reason for this difference may be the isoelectric points (pH values of zero charge) of *E*. *coli* KO11 and MS2 [27]. The isoelectric points of *E*. *coli* KO11 and MS2 are 5.6 and 3.5–3.9, respectively [28, 29]. Because the average pH of the test water was 7.6±0.29 (S1 Table in S1 File), the lower isoelectric point of MS2 would cause it to have a larger negative charge in the test water near pH neutral, as compared to *E*. *coli* KO11. Due to this difference in magnitude of surface charge, MS2 may have a stronger electrostatic attraction with the positively-charged chitosan acetate coagulant than *E*. *coli*, resulting in a higher LRVs from chitosan pre-treatment alone.

Charge neutralization, which occurs when negatively charged particles such as viruses, bacteria, silt, clay, and other organic and inorganic matter, adsorb onto positively charged sites of the chitosan polymeric chain are thought to contribute to coagulation-flocculation processes, with progressively larger flocs formed as this process occurs repeatedly. Flocs that are large and dense enough will then sediment due to gravity, reducing viral, bacterial, and colloidal particles in the supernatant water and facilitate their removal by filtration processes. Although these mechanisms are proposed in the available literature, the physio-chemical interactions between chitosan and microbes in coagulation-flocculation and cloth filtration are not yet well described [30–32].

Another reason for the difference in LRVs between MS2 coliphage and *E*. *coli* KO11 may have been due to differences in their physical sizes and surface-area-to-mass-ratios. Smaller suspended particles like viruses, which are about 40 times smaller in diameter than individual *E*. *coli* cells, have a higher surface-area-to-mass-ratio, than larger particles like bacteria, which have a lower-surface area-to-mass-ratio. Both *E*. *coli* and MS2 would have negative surface charges in the test water. However, because MS2 is smaller and has a larger surface-area-to-mass-ratio, the negative surface charge may play a larger role in its mobility and interaction with other constituents in the water, and possibly make it more effectively removable by chitosan coagulation, as compared to *E*. *coli* KO11 [33].

Hydrophobic interactions may also have influenced MS2 and *E*. *coli* interactions with chitosan. While chitosan has a net positive charge when dissolved in water, the linear polysaccharide is actually amphiphilic, allowing hydrophobic interactions by its neutral and positively charged sugar units. The neutral units (GlcNAc), or A-units, are responsible for the hydrophobic effects and contain a rather bulky acetyl group. The charged units (GlcN), or D-units, are responsible for the hydrophilic/electrostatic interactions and contain a positively charged amino group (-NH_3_^+^) produced by deacetylation and protonation. Chitosan’s hydrophobicity depends on the chain length and the ratio of A-units to D-units, which depends on the degree of deacetylation [34]. Virus and bacteria surfaces often have both hydrophobic and hydrophilic surfaces with some viral surfaces being more hydrophobic than others. *E*. *coli* has been characterized for its surface functional groups responsible for electrical charge and hydrophobicity, although not specifically for effects on specific interactions with different particles or surfaces [35]. For the two similar *Leviviridae* family members, MS2 and Q-Beta, the surface of the former has more polar characteristics as compared to that of the latter, which has more apolar characteristics and is therefore more responsive to hydrophobic interactions [36]. Therefore, hydrophobic effects may play a role in the coagulation process when there is viral and bacterial adsorption to hydrophobic acetyl regions (A-units) of the chitosan chain. As amino groups on the D-units deprotonate at higher pH levels, hydrophobic interactions become more dominant as compared to electrostatic and ionic forces [34, 36–38]. In natural waters with negatively charged particles such as bacteria, viruses, protozoans, clay, and silt, and having pH levels ranging from 5 to 9 [39], the hydrophobic effects of chitosan may play a greater role in more basic natural waters with higher pH. E.M. van Voorthuizen et al., 2001 found that hydrophobic interactions play an important role in the adsorption and retention of MS2 coliphage on filters [40]. There is also evidence suggesting that above the pI of a particular virus, hydrophobic effects may be more important for adsorption than previously thought [40]. The effects of hydrophobicity on virus and bacteria adsorption and coagulation-flocculation were not specifically studied in the current work, but is a possible explanation for the higher observed MS2 coliphage LRVs than those of *E*. *coli*. Both charge neutralization and inter-particle bridging are considered the two major mechanisms by which chitosan is thought to work.

While we have speculated some potential mechanisms (i.e., differences in size, electrical potential, and hydrophobicity), the observed greater removal of MS2 compared to *E*. *coli* by chitosan pre-treatment is not something we can fully explain without further exploration. All of these discussed factors can influence the extent of microbial reduction by chitosan pre-treatment, but they were not systematically explored in this current study due to limitations of time and other resources.

In communities where CWFs or sand filters are already utilized, cloth filtration may not be a preferred filtration treatment because these more robust filtration technologies are already implemented. However, chitosan pre-treatment may be well partnered with CWFs and sand filters to improve the effectiveness of these filtration technologies to reduce microbial contaminants and turbidity while reducing their clogging and backwashing frequencies. Where cloth/saree filters are already commonly used, chitosan pre-treatment could be implemented as an attractive, achievable and cost-effective filtration option because filtration time is relatively short and filters are made of scrap cloth that is already available from the household; hence, there is no need to purchase a new filter technology. Cloth filters do require cleaning prior to reuse, typically by washing them, while CWFs and BioSand filters must be cleaned periodically by scouring to remove accumulated particulate contaminants. However, unlike CWFs and BSFs, cloth filters do not require specific water collection containers, they cannot crack like CWFs, and they do not require the periodic scouring required of CWFs or BioSand filters to restore flow rates. The cloth filtration apparatus can be adjusted to the water filtration preferences of the user.

Because coagulation may be more effective for microbes that have a lower pI or larger diameter, it is important to test the effectiveness of coagulation pre-treatment and filtration with microbes that have a range of characteristics. Coliphage MS2, with a diameter of 22.4–28.8 nm and a pI of 3.5–3.9, is smaller in size as compared to many other viruses that range from about 20 nm to 85 μm in size [41, 42]. Bacteriophage Qβ has a higher pI of 5.3 but is similar in diameter to MS2 at approximately 21.3–29.4 nm, while bacteriophage PRD1 has a pI of 4.2 and diameter of ~66 nm [43]. These viruses as well as bacteria like *E*. *coli* could be used to further evaluate the effects of bacteriophage and bacteria pI, hydrophobicity and size on chitosan coagulation and cloth filtration processes [44, 45].

LRVs may have been over or under estimated due to direct aggregation or “clumping” of organisms that bind together, sometimes, preventing a homogenous distribution of viral and bacterial particles throughout the effluent water. In this study the effects of direct virus and bacteria aggregation in the absence of chitosan coagulation-flocculation pre-treatment were not specifically investigated but are considered minimal because MS2 and *E*. *coli* LRVs by direct filtration were relatively low (<0.5), compared to the much greater reductions by chitosan coagulation-flocculation pre-treatment.

While intermediate stirring conditions resulted in significantly higher LRVs for *E*. *coli* and higher, but non-significant, LRVs for MS2, floc size from particle size analysis indicated smaller floc size formation with the use of the intermediate stirring than the standard stirring condition. This inconsistency in results may have been due to limitations in the apparatus set-up of the Mastersizer analyzer. Challenge water was pushed through the Mastersizer using a peristaltic pump, which was required to collect the samples. The pumping action caused a physical disturbance in the water, similar to the motion of mixing the water. Pumping and the transfer in and out of the tubing may have caused fragmentation of the formed flocs, resulting in measured floc sizes that were much lower than actual floc sizes for all experiments. Each stirring procedure was associated with varying mixing and settling times; therefore, floc formation during each stirring procedure may have been affected differently. Although particle size analysis may not have revealed true floc size formation in microbial experiments, relative floc sizes determined among challenge water types and stirring conditions is still useful information that helps explain floc size as a factor in microbial reduction by sedimentation and filtration.

The effects of water quality on the effectiveness of chitosan as a coagulant was not specifically or systematically studied. A convenient and representative sample water was used for all microbial experiments, with two different water sampling times for particle size analysis of flocs with the Mastersizer 3000 by Malvern. Water sampled in March 2019, with a lower alkalinity and TOC, gave significantly lower floc size formation among all three stirring conditions compared to the water collected in August. While water quality was not directly studied, it is speculated that water quality parameters, such as turbidity, alkalinity, pH, dissolved solids, hardness and TOC, may have an effect on chitosan’s ability to form larger flocs. Seasonal differences in water quality and temperature of challenge water were not specifically studied here but may also impact chitosan acetate coagulation-flocculation and filtration performance and should be further investigated in future studies.

## Conclusions

The aim of this research was to evaluate the potential to improve microbial reductions with the use of chitosan pre-treatment by coagulation-flocculation prior to cloth filtration for point-of-use HWT. The WHO HWT performance tiers for bacterial and viral log_10_ reductions were used as the basis for microbial reduction performance. Chitosan acetate coagulation-flocculation-sedimentation, at a 10 mg/L dose, followed by 12-layers of cloth filtration, was able to achieve the WHO 2-star protective performance level (>3-Log_10_ virus reductions and >2-Log_10_ bacteria reductions) and reduce effluent turbidity to 1 NTU or less. This performance level is likely to reduce health risks in comparison to filtration through these cloth filters alone, which gave <1 log_10_ reductions that did not achieve even the WHO 1-star performance level.

Chitosan floc particle size analysis resulted in the standard stirring conditions producing the largest floc sizes for both challenge waters, but intermediate stirring conditions resulted in *E*. *coli* KO11 reductions that were significantly higher than standard and minimal stirring conditions. Intermediate stirring conditions also resulted in higher reductions in MS2 and turbidity, although not statistically significant.

Despite the expressed limitations of this study and the need for future work, this research demonstrates the ability of chitosan acetate pre-treatment by coagulation-flocculation-sedimentation to significantly improve the microbial reduction performance of cloth filtration as a POU water treatment technology. Further optimization of this technology is recommended as future work in both lab and field studies.

## Supporting information

S1 File (DOCX)
