# Supplementary material for: Microbial reductions and physical characterization of chitosan flocs when using chitosan acetate as a cloth filter aid in water treatment
Source: PLoS One. 2022 Jan 21;17(1):e0262341. doi: 10.1371/journal.pone.0262341 (PMC8782320; doi:10.1371/journal.pone.0262341)
Supplement: S1 File — (DOCX) [file pone.0262341.s001.docx]

**Supporting Information: Microbial Reductions and Physical Characterization of Chitosan Flocs when Using Chitosan Acetate as a Cloth Filter Aid**

Hemali H. Oza^1,2^*, Eleanor B. Holmes^2^, Emily S. Bailey^3^, Collin K. Coleman^2^, Mark D. Sobsey^2^

^1^ Gangarosa Department of Environmental Health, Rollins School of Public Health, Emory University, Atlanta, GA 30033, USA

^2^ Department of Environmental Sciences and Engineering, Gillings School of Global Public Health, University of North Carolina, Chapel Hill, NC 27599, USA

^3^ Texas Tech University Health Sciences Center, Graduate School of Biomedical Sciences, Julia Jones Matthews Department of Public Health, Abilene, TX 79601, USA

* Correspondence: hemali.harish.oza@emory.edu; Telephone: +1 (919) 561-2233

**S1 Table. Quality parameters and values for raw University Lake water samples used in experiments.^a^**

**S2 Table. Parameter conditions for microbial experiments.**

**S3 Table. Three stirring conditions used, standard, intermediate, and minimal, were defined by various mixing and settling conditions.**

**S4 Table. Parameter conditions for particle size analysis experiments.**

**S5 Table. Mastersizer 3000 by Malvern settings for chitosan acetate coagulation-flocculation and sedimentation experiments using three stirring conditions.**

**S6 Table. Turbidity values for each sampling point by stirring condition and test water.**

**Table S7. LRVs and associated 95% confidence intervals for *E. coli* KO11 and MS2 coliphage per challenge water type, stirring condition, and various points of water sampling, in waters with and without chitosan acetate pre-treatment.**

**S1 Fig.** **100% cotton cloth filter material under a light microscope.** New (boxes A, C, E) and used (boxes B, D, F) cloth filter material were observed under a light microscope to measure the approximate pore size (~100 μm). The fibers of the used cloth are visibly agitated and frayed (box F), as compared to the new material (box E).

**S8 Table. Comparisons of estimated mean differences in average LRVs of *E. coli* KO11 and MS2 coliphage after controlling for selected parameters in linear regressions.**

**S9 Table. Average floc size and 95% confidence limits for the last 30 data points from each triplicate experiment per challenge water type and stirring condition.**

**S10 Table. T-tests for statistical significance between pairs of test water with and without added sewage and different mixing conditions during coagulation-flocculation and settling.^6^**

**S11 Table. pH values for each sampling point by stirring condition and test water.**

**S12 Table. Raw log_10_ reduction values foe *E. coli* KO11 and MS2 coliphage.**

**S13 Table. Raw floc size data by water sample and stirring condition.**

**S1 Table.** Quality parameters and values for raw University Lake water samples used in experiments^a^

| Parameter | Units | Average ± 95% CI | |
| --- | --- | --- | --- |
|  |  | Sampled August 18, 2018 | Sampled March 1, 2019 |
| Turbidity | NTU | 6.21±0.72 | 23.6±0.32 |
| pH | - | 7.57±0.29 | 6.89±0.02 |
| Alkalinity^b^ | Mg/L CaCO3 | 30 | 17.167±0.32 |
| Total Organic Content | mg/L | 8.81 | 5.94 |
| Dissolved Oxygen Content | mg/L | 7.67 | 5.19 |
| UV254 | cm-1 | 0.242 | 0.198 |
| Total Coliform | MPN/100 mL | 408 | 308 |
| *E. coli* | MPN/100 mL | <4 | 50 |
| Specific Conductance | μmohs/cm | 87 | 65.5 |
| Fluoride | mg/L | <0.10 | <0.10 |
| Manganese | mg/L | 0.282 | 0.095 |
| Iron | mg/L | 0.91 | 0.53 |
| Hardness | mg/L CaCO3 | 31±1.39 | 20 |

^a^ Water for microbial experiments and for particle size analysis of test water with no added sewage was sampled on August 18, 2019. Water used for particle size analysis for sewage-amended test water was sampled on March 1, 2019. Water quality parameter measurements listed in the table are prior to the addition of sewage and reflect the quality of the raw sampled water. Water quality measurements were reported by OWASA.

^b^ OWASA measures alkalinity as a raw water blend of 25% University Lake and 75% Cane Creek Reservoir (another nearby surface water reservoir).

**S2 Table.** Parameter conditions for microbial experiments

| Filtration after Chitosan  Pre-treatment | Test Microorganisms | Challenge Water (August collection) | Chitosan Acetate Dose | Stirring conditions | Replicates |
| --- | --- | --- | --- | --- | --- |
| 12-layers of 100% cotton cloth | *E. coli* KO11  MS2 coliphage | Natural Lake Water  (August 18, 2018) | 0 mg/L | NA | x3 |
|  |  |  | 10 mg/L | Standard |  |
|  |  |  |  | Intermediate |  |
|  |  |  |  | Minimal |  |
|  |  | Natural Lake Water  +1% Pasteurized Sewage  (August 18, 2018) | 0 mg/L | NA |  |
|  |  |  | 10 mg/L | Standard |  |
|  |  |  |  | Intermediate |  |
|  |  |  |  | Minimal |  |

**S3 Table.** Three stirring conditions used, standard, intermediate, and minimal, were defined by various mixing and settling conditions.

| Stirring Condition | Duration (minutes) | Mixing Speed (RPM) |
| --- | --- | --- |
| Standard | 1 | 100 |
|  | 15 | 25 |
|  | 30 | 0 |
| Intermediate | 1 | 100 |
|  | 2 | 25 |
|  | 5 | 0 |
|  | 2 | 25 |
|  | 5 | 0 |
|  | 2 | 25 |
|  | 30 | 0 |
| Minimal | 1 | 100 |
|  | 30 | 0 |

Stirring conditions varied depending on the experimental condition; controls were not stirred. Three different stirring conditions were chosen to evaluate three different types of mixing. The standard condition is a standard coagulation-flocculation and sedimentation procedures that is robust but not as practical in a field setting due to the need for continuous slow mixing for 15 minutes. A minimal stirring condition, which may be more practical in a field setting but may not facilitate proper floc formation, was just one minute of rapid stirring. An intermediate stirring condition of periodic mixing of two minutes each followed by five minutes of no mixing, a total of two times and then a two-minute mix followed by 30 minutes of settling, was used to better facilitate floc formation while still being practical in a field setting.

**S4 Table.** Parameter conditions for particle size analysis experiments

| Chitosan Acetate Dose | Challenge Water | Stirring conditions *(triplicate samples)* | Replicates |
| --- | --- | --- | --- |
| 10 mg/L | Natural Lake Water  (August 18, 2018) | Standard | x3 |
|  |  | Intermediate |  |
|  |  | Minimal |  |
|  | Natural Lake Water  + 1% Pasteurized Sewage  (March 1, 2019) | Standard |  |
|  |  | Intermediate |  |
|  |  | Minimal |  |

**S5 Table.** Mastersizer 3000 by Malvern settings for chitosan acetate coagulation-flocculation and sedimentation experiments using three stirring conditions.

| Parameter | | Setting | | |
| --- | --- | --- | --- | --- |
| Identification | Material Name | Oleic Acid (approximate equivalent) | | |
|  | Particle Type | Non-Spherical | | |
|  | Refractive Index | 1.433 | | |
|  | Absorption Index | 0.001 | | |
|  | Density (g/cm^3^) | 1.002 | | |
| Dispersant | Dispersant Name | Water | | |
|  | Refractive Index | 1.33 | | |
| Duration  (seconds, s) | Stirring Procedure | Standard | Intermediate | Minimal |
|  | Red Background measurement (s) | 10 | 10 | 10 |
|  | Red Sample measurement (s) | 10 | 10 | 10 |
|  | Blue Background measurement (s) | 5 | 5 | 5 |
|  | Blue Sample Measurement duration (s) | 5 | 5 | 5 |
|  | Number of measurements | 64 | 64 | 64 |
|  | Delay between measurements (s) | 13 | 14 | 0 |
|  | Pre-measurement delay (s) | 0 | 0 | 0 |
| Obscuration | Obscuration Lower Limit (%) | 2 | | |
|  | Obscuration Upper Limit (%) | 20 | | |

**S6 Table.** Turbidity values for each sampling point by stirring condition and test water.

| Test Water | Stirring Conditions | Raw Turbidity Values (NTU) | | | | |
| --- | --- | --- | --- | --- | --- | --- |
|  |  | Raw Lake Water | Influent | Filtration Alone | Pre-treated | Effluent |
| No Pasteurized Sewage | Standard | 3.6 | 3.6 | 2.5 | 0.90 | 0.31 |
|  |  |  |  |  |  | 0.25 |
|  |  |  |  |  |  | 0.30 |
|  | Intermediate | 8.6 | 11.3 | 2.5 | 3.7 | 0.53 |
|  |  |  |  |  |  | 0.47 |
|  |  |  |  |  |  | 0.48 |
|  | Minimal | 7.1 | 7.4 | 6.2 | 6.4 | 0.81 |
|  |  |  |  |  |  | 0.90 |
|  |  |  |  |  |  | 0.79 |
| +1% Pasteurized Sewage | Standard | 5.0 | 7.2 | 2.3 | 1.4 | 0.80 |
|  |  |  |  |  |  | 0.35 |
|  |  |  |  |  |  | 0.40 |
|  | Intermediate | 5.9 | 7.6 | 3.3 | 3.2 | 0.71 |
|  |  |  |  |  |  | 0.30 |
|  |  |  |  |  |  | 0.55 |
|  | Minimal | 7.1 | 8.7 | 7.6 | 6.4 | 0.66 |
|  |  |  |  |  |  | 0.85 |
|  |  |  |  |  |  | 0.52 |

**S7** **Table.** LRVs and associated 95% confidence intervals for *E. coli* KO11 and MS2 coliphage per challenge water type, stirring condition, and various points of water sampling, in waters with and without chitosan acetate pre-treatment. See S12 Table for full raw data.

| Challenge Water Type | Stirring Condition | Treatment Sections^d^ | LRV ± 95% Confidence Interval | |
| --- | --- | --- | --- | --- |
|  |  |  | *E. coli* KO11 | MS2 Coliphage |
| Non-past. Sewage amended | Filtration Alone | | 0.10 ± 0.03 | 0.10 ± 0.030 |
|  | Standard^3^ | Post CH | 1.21 ± 0.27 | 1.68 ± 0.28 |
|  |  | EF-CH | 1.87 ± 0.32 | 1.38 ± 0.61 |
|  |  | Effluent | 3.11 ± 0.31 | 3.22 ± 0.61 |
|  | Intermediate^c^ | Post CH | 1.17 ± 0.22 | 1.54 ± 0.20 |
|  |  | EF-CH | 2.41 ± 0.77 | 1.87 ± 0.34 |
|  |  | Effluent | 3.57 ± 0.77 | 3.41 ± 0.15 |
|  | Minimal^3^ | Post CH | 0.78 ± 0.20 | 1.77 ± 0.36 |
|  |  | EF-CH | 2.33 ± 0.48 | 1.17 ± 0.37 |
|  |  | Effluent | 3.16 ± 0.48 | 3.15 ± 0.37 |
| 1% past. Sewage amended | Filtration Alone | | 0.12 ± 0.11 | 0.35 ± 0.28 |
|  | Standard^3^ | Post CH | 1.27 ± 0.34 | 2.36 ± 0.30 |
|  |  | EF-CH | 2.07 ± 0.26 | 1.34 ± 0.28 |
|  |  | Effluent | 3.43 ± 0.26 | 3.73 ± 0.28 |
|  | Intermediate^3^ | Post CH | 1.36 ± 0.25 | 2.43 ± 0.24 |
|  |  | EF-CH | 2.54 ± 0.50 | 1.14 ± 0.29 |
|  |  | Effluent | 4.09 ± 0.50 | 3.64 ± 0.29 |
|  | Minimal^3^ | Post CH | 0.93 ± 0.13 | 1.28 ± 0.25 |
|  |  | EF-CH | 2.28 ± 0.57 | 2.33 ± 0.32 |
|  |  | Effluent | 3.20 ± 0.57 | 3.47 ± 0.32 |

^c^ Dosed with 10 mg/L of chitosan acetate pre-treatment using the associated stirring condition

^d^ Post CH = reductions due to chitosan pre-treatment alone; EF-CH = reductions due to pre-treatment and filtration minus reductions due to pre-treatment alone; Effluent = reductions due to pre-treatment and filtration together


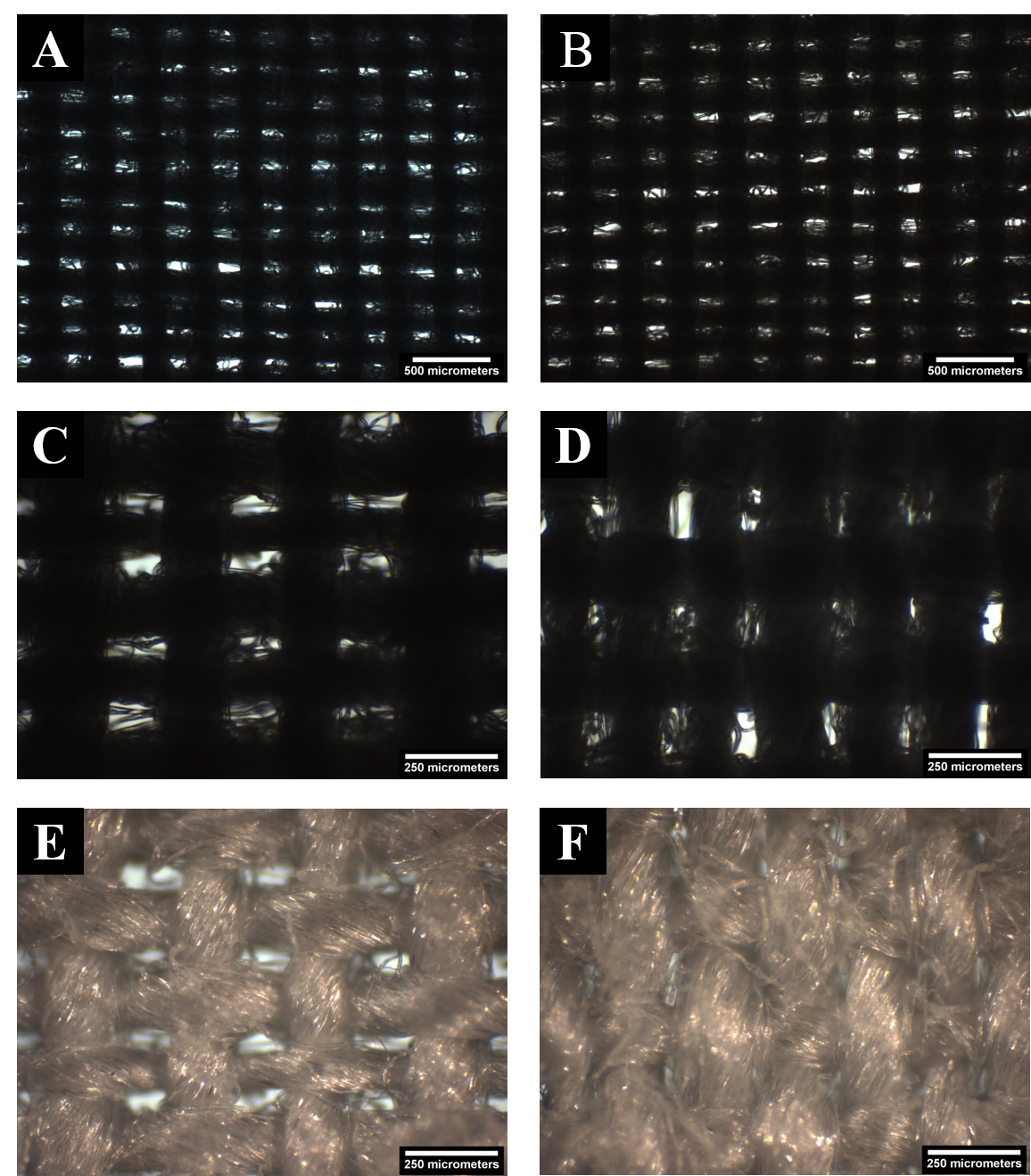


**S1 Fig.** 100% cotton cloth filter material under a light microscope; new (boxes A, C, E) and used (boxes B, D, F) cloth filter material were observed under a light microscope to measure the approximate pore size (~100 μm). The fibers of the used cloth are visibly agitated and frayed (box F), as compared to the new material (box E).

**S8 Table.** Comparisons of estimated mean differences in average LRVs of *E. coli* KO11 and MS2 coliphage after controlling for selected parameters in linear regressions. See S12 Table for full raw data.

|  | Comparison Parameters | | | Estimated LRV Mean Difference | Standard Error | Pr(>\|t\|) |
| --- | --- | --- | --- | --- | --- | --- |
|  | LRV_A_ > LRV_B_ | | |  |  |  |
| *E. coli* KO11 | Water Sample | | |  |  |  |
|  |  | Sewage | No Sewage | 0.14 | 0.080 | 0.094’ |
|  |  |  |  |  |  |  |
|  | Stirring Conditions | | |  |  |  |
|  |  | Intermediate | Minimal | 0.31 | 0.10 | 0.0024** |
|  |  | Intermediate | Standard | 0.28 | 0.10 | 0.0064** |
|  |  | Standard | Minimal | 0.03 | 0.10 | 0.74 |
|  |  |  |  |  |  |  |
|  | Treatment Stage | | |  |  |  |
|  |  | CHEF | Pre-treatment Alone | 1.13 | 0.11 | < 1.0 × 10^-5^ *** |
|  |  | Pre-treatment & Filtration | Pre-treatment Alone | 2.30 | 0.11 | < 1.0 × 10^-5^ *** |
|  |  | Pre-treatment | Filtration Alone | 1.01 | 0.11 | < 1.0 × 10^-5^ *** |
|  |  | Pre-treatment & Filtration | CHEF | 1.18 | 0.11 | < 1.0 × 10^-5^ *** |
|  |  | CHEF | Filtration Alone | 2.14 | 0.11 | < 1.0 × 10^-5^ *** |
|  |  | Pre-treatment & Filtration | Filtration Alone | 3.32 | 0.11 | < 1.0 × 10^-5^ *** |
| MS2 coliphage | Water Sample | | |  |  |  |
|  |  | Sewage | No Sewage | 0.27 | 0.090 | 0.0040** |
|  |  |  |  |  |  |  |
|  | Stirring Conditions | | |  |  |  |
|  |  | Intermediate | Minimal | 0.11 | 0.11 | 0.34 |
|  |  | Intermediate | Standard | 0.04 | 0.11 | 0.73 |
|  |  | Standard | Minimal | 0.07 | 0.11 | 0.54 |
|  |  |  |  |  |  |  |
|  | Treatment Stage | | |  |  |  |
|  |  | Pre-treatment Alone | CHEF | 0.30 | 0.13 | 0.022* |
|  |  | Pre-treatment & Filtration | Pre-treatment Alone | 1.59 | 0.13 | < 1.0 × 10^-5^ *** |
|  |  | Pre-treatment Alone | Filtration Alone | 1.61 | 0.13 | < 1.0 × 10^-5^ *** |
|  |  | Pre-treatment & Filtration | CHEF | 1.90 | 0.13 | < 1.0 × 10^-5^ *** |
|  |  | CHEF | Filtration Alone | 1.31 | 0.13 | < 1.0 × 10^-5^ *** |
|  |  | Pre-treatment & Filtration | Filtration Alone | 3.21 | 0.13 | < 1.0 × 10^-5^ *** |
| Post Chitosan |  | MS2 | *E. coli* KO11 | 0.72 | 0.117 | < 0.000010*** |

Using linear regressions, the estimated LRV mean difference was calculated for three parameters—water sample type, stirring conditions, and treatment stages. After controlling for stirring conditions and treatment stages, estimated LRV mean differences among water sample types were calculated for both microbes, separately. Second, after controlling for water sample types and treatment stages, estimated LRV mean differences among water samples were calculated for both microbes, separately. *CHEF* refers to the isolated effects of filtration after pre-treatment (LRV_Pre-treatment & Filtration_ - LRV_Pre-treatment Alone_). Third, after controlling for water sample types and stirring conditions, estimated LRV mean differences among treatment stages were calculated for both microbes, separately. Lastly, after controlling for water sample type and stirring conditions, estimated LRV mean differences among MS2 coliphage and *E. coli* KO11 were calculated for pre-treated samples.

‘α = 0.10; 90% confidence-level

* α = 0.050; 95% confidence-level

** α = 0.010; 99% confidence-level

*** α < 1.0 × 10^-5^; > 99.999% confidence-level

**S9 Table.** Average floc size and 95% confidence limits for the last 30 data points from each triplicate experiment per challenge water type and stirring condition. See S13 Table for full raw data.

| Challenge Water Type | Stirring Condition | Average Floc Size  ± 95% Confidence Interval (µM) |
| --- | --- | --- |
| Non-Sewage-amended Water  (Sampled August) | Standard | 248±27 |
|  | Intermediate | 129±3.0 |
|  | Minimal | 150±3.2 |
| 1% Pasteurized-sewage-amended Water  (Sampled March) | Standard | 91±1.5 |
|  | Intermediate | 61±7.0 |
|  | Minimal | 80±2.9 |

**S10 Table.** T-tests for statistical significance between pairs of test water with and without added sewage and different mixing conditions during coagulation-flocculation and settling.^e^ See S13 Table for full raw data.

|  |  | Comparison | | 95% Confidence Interval of Mean Difference in Floc Particle Size (µM) | p-value |
| --- | --- | --- | --- | --- | --- |
| Non-sewage amended (August) | > | Sewage amended (March) | Standard | 129.46, 184.87 | < 1.0x10^-5 †^ |
|  |  |  | Intermediate | 60.28, 75.71 | < 1.0x10^-5^ ^†^ |
|  |  |  | Minimal | 65.61, 74.21 | < 1.0x10^-5^ ^†^ |
| Non-sewage amended (August) | | | Standard > Intermediate | 90.84, 146.49 | < 1.0x10^-5^ ^†^ |
|  |  |  | Standard > Minimal | 70.34, 126.02 | < 1.0x10^-5^ ^†^ |
|  |  |  | Minimal > Intermediate | 16.09, 24.89 | < 1.0x10^-5^ ^†^ |
| Sewage amended (March) | | | Standard > Intermediate | 22.24, 36.75 | < 1.0x10^-5^ ^†^ |
|  |  |  | Standard > Minimal | 7.66, 14.17 | < 1.0x10^-5^ ^†^ |
|  |  |  | Minimal > Intermediate | 79.96, 61.38 | < 1.0x10^-5^ ^†^ |

^e^ The last 30 measurement points from each triplicate particle size analysis experiment were combined for a total sample size of 90 points per stirring condition and water sample type. Non-sewage PSA experiments were conducted with water sampled on August 18^th^, 2019, and sewage-amended PSA experiments were conducted with water sampled on March 1^st^, 2019. Combined sample points for non-sewage amended samples and sewage amended samples of the same stirring conditions were compared, as well as comparisons of different stirring conditions for the same challenge water samples, using a t-test for parametric analysis. The 95% confidence interval of mean difference among the two compared data sets, the t-value, and the associated p-values.

^†^ α = 0.00001; 99.999% confidence-level

**S11 Table.** pH values for each sampling point by stirring condition and test water.

| Test Water | Stirring Conditions | Raw pH Values | | | | |
| --- | --- | --- | --- | --- | --- | --- |
|  |  | Raw Lake Water | Influent | Filtration Alone | Pre-treated | Effluent |
| No Pasteurized Sewage | Standard | 7.1 | 7.0 | 7.4 | 7.0 | 7.0 |
|  |  |  |  |  |  | 7.4 |
|  |  |  |  |  |  | 7.7 |
|  | Intermediate | 8.9 | 8.7 | 9.0 | 8.8 | 8.5 |
|  |  |  |  |  |  | 8.5 |
|  |  |  |  |  |  | 8.6 |
|  | Minimal | 7.2 | 7.2 | 7.5 | 7.2 | 7.3 |
|  |  |  |  |  |  | 7.5 |
|  |  |  |  |  |  | 7.3 |
| +1% Pasteurized Sewage | Standard | 7.5 | 7.5 | 7.6 | 7.5 | 7.6 |
|  |  |  |  |  |  | 7.8 |
|  |  |  |  |  |  | 8.4 |
|  | Intermediate | 7.4 | 7.4 | 7.7 | 7.5 | 7.8 |
|  |  |  |  |  |  | 7.7 |
|  |  |  |  |  |  | 8.1 |
|  | Minimal | 7.2 | 7.2 | 7.3 | 7.3 | 7.6 |
|  |  |  |  |  |  | 7.3 |
|  |  |  |  |  |  | 7.4 |

**S12 Table. Raw log_10_ reduction values foe *E. coli* KO11 and MS2 coliphage.**

| Water Sample Type | Stirring Condition | Sample Point | Rep. 1 | | Rep. 2 | Rep 3. | Average | n | 95% CI |
| --- | --- | --- | --- | --- | --- | --- | --- | --- | --- |
| *E. coli* KO11 | | | | | | | | | |
| No Sewage | Filtration Alone | | | 0.124 | 0.093 | 0.075 | 0.098 | 3 | 0.028 |
|  | Standard | Post CH | | 1.241 | 0.959 | 1.426 | 1.209 | 3 | 0.267 |
|  |  | CH to EF | | 2.099 | 1.942 | 1.555 | 1.865 | 3 | 0.317 |
|  |  | Effluent | | 3.340 | 3.183 | 2.796 | 3.106 | 3 | 0.317 |
|  | Intermediate | Post CH | | 1.165 | 1.378 | 0.981 | 1.175 | 3 | 0.225 |
|  |  | CH to EF | | 2.108 | 3.187 | 1.932 | 2.409 | 3 | 0.769 |
|  |  | Effluent | | 3.273 | 4.353 | 3.097 | 3.574 | 3 | 0.769 |
|  | Minimal | Post CH | | 0.836 | 0.930 | 0.586 | 0.784 | 3 | 0.201 |
|  |  | CH to EF | | 2.472 | 1.848 | 2.659 | 2.327 | 3 | 0.481 |
|  |  | Effluent | | 3.309 | 2.684 | 3.496 | 3.163 | 3 | 0.481 |
| Sewage | Filtration Alone | | | 0.028 | 0.229 | 0.104 | 0.120 | 3 | 0.115 |
|  | Standard | Post CH | | 1.359 | 1.513 | 0.937 | 1.270 | 3 | 0.337 |
|  |  | CH to EF | | 2.269 | 2.116 | 1.821 | 2.069 | 3 | 0.257 |
|  |  | Effluent | | 3.628 | 3.475 | 3.181 | 3.428 | 3 | 0.257 |
|  | Intermediate | Post CH | | 1.549 | 1.122 | 1.409 | 1.360 | 3 | 0.247 |
|  |  | CH to EF | | 2.060 | 2.935 | 2.634 | 2.543 | 3 | 0.503 |
|  |  | Effluent | | 3.610 | 4.485 | 4.184 | 4.093 | 3 | 0.503 |
|  | Minimal | Post CH | | 0.914 | 0.828 | 1.062 | 0.935 | 3 | 0.134 |
|  |  | CH to EF | | 2.429 | 1.724 | 2.690 | 2.281 | 3 | 0.565 |
|  |  | Effluent | | 3.344 | 2.638 | 3.604 | 3.195 | 3 | 0.565 |
| MS2 Bacteriophage | | | | | | | | | |
| No Sewage | Filtration Alone | | | 0.099 | 0.133 | 0.081 | 0.104 | 3 | 0.030 |
|  | Standard | Post CH | | 1.833 | 1.398 | 1.814 | 1.682 | 3 | 0.278 |
|  |  | CH to EF | | 1.934 | 0.849 | 1.380 | 1.388 | 3 | 0.614 |
|  |  | Effluent | | 3.767 | 2.682 | 3.213 | 3.220 | 3 | 0.614 |
|  | Intermediate | Post CH | | 1.674 | 1.598 | 1.338 | 1.537 | 3 | 0.200 |
|  |  | CH to EF | | 1.707 | 1.697 | 2.220 | 1.875 | 3 | 0.338 |
|  |  | Effluent | | 3.382 | 3.294 | 3.558 | 3.411 | 3 | 0.152 |
|  | Minimal | Post CH | | 1.979 | 1.921 | 1.405 | 1.769 | 3 | 0.358 |
|  |  | CH to EF | | 1.276 | 0.808 | 1.427 | 1.170 | 3 | 0.365 |
|  |  | Effluent | | 3.255 | 2.787 | 3.406 | 3.150 | 3 | 0.365 |
| Sewage | Filtration Alone | | | 0.423 | 0.562 | 0.079 | 0.355 | 3 | 0.282 |
|  | Standard | Post CH | | 2.396 | 2.599 | 2.081 | 2.359 | 3 | 0.295 |
|  |  | CH to EF | | 1.273 | 1.606 | 1.132 | 1.337 | 3 | 0.275 |
|  |  | Effluent | | 3.669 | 4.003 | 3.529 | 3.734 | 3 | 0.275 |
|  | Intermediate | Post CH | | 2.494 | 2.195 | 2.605 | 2.431 | 3 | 0.240 |
|  |  | CH to EF | | 1.002 | 1.441 | 0.983 | 1.142 | 3 | 0.294 |
|  |  | Effluent | | 3.496 | 3.935 | 3.477 | 3.636 | 3 | 0.294 |
|  | Minimal | Post CH | | 1.146 | 1.531 | 1.160 | 1.279 | 3 | 0.247 |
|  |  | CH to EF | | 2.368 | 2.589 | 2.028 | 2.328 | 3 | 0.320 |
|  |  | Effluent | | 3.514 | 3.734 | 3.173 | 3.474 | 3 | 0.320 |

**S13 Table. Raw floc size data by water sample and stirring condition.^f^**

|  | Floc Size Measures (µM) | | | | | |
| --- | --- | --- | --- | --- | --- | --- |
| Sample Type | No-sewage amended samples | | | Sewage amended samples | | |
| Stirring Condition | Standard | Intermediate | Minimal | Standard | Intermediate | Minimal |
| From 1^st^ replicate | 431 | 141 | 146 | 105 | 90.6 | 76.8 |
|  | 439 | 139 | 145 | 105 | 90.6 | 76.7 |
|  | 455 | 137 | 145 | 104 | 90.7 | 76.5 |
|  | 448 | 136 | 145 | 103 | 90.1 | 76.7 |
|  | 438 | 136 | 145 | 103 | 90.2 | 76.4 |
|  | 435 | 134 | 144 | 102 | 90.4 | 76.8 |
|  | 445 | 132 | 144 | 101 | 90.2 | 76.6 |
|  | 442 | 133 | 144 | 101 | 90.3 | 76.5 |
|  | 442 | 131 | 144 | 100 | 89.8 | 76.5 |
|  | 437 | 131 | 145 | 99.6 | 89.6 | 76.4 |
|  | 440 | 129 | 144 | 99.6 | 89.8 | 76.6 |
|  | 427 | 129 | 143 | 99 | 89.7 | 76.2 |
|  | 438 | 128 | 144 | 98.7 | 89.6 | 76.3 |
|  | 432 | 126 | 143 | 98.4 | 89.4 | 76.2 |
|  | 429 | 126 | 144 | 97.8 | 89.4 | 75.8 |
|  | 434 | 124 | 144 | 97.6 | 89.3 | 75.7 |
|  | 420 | 124 | 143 | 97.1 | 89.2 | 75.5 |
|  | 420 | 124 | 144 | 96.4 | 89 | 75.7 |
|  | 422 | 123 | 135 | 96.1 | 89.2 | 75.5 |
|  | 437 | 122 | 133 | 95.6 | 89.1 | 75.3 |
|  | 428 | 122 | 130 | 95.5 | 88.9 | 75.3 |
|  | 422 | 121 | 128 | 95.5 | 88.6 | 74.7 |
|  | 412 | 120 | 126 | 95 | 88.7 | 75.1 |
|  | 409 | 118 | 124 | 94.6 | 88.4 | 74.8 |
|  | 396 | 118 | 123 | 93.5 | 88.4 | 74.9 |
|  | 399 | 119 | 121 | 93.3 | 88.4 | 74.8 |
|  | 396 | 117 | 119 | 93 | 88.2 | 74.3 |
|  | 413 | 117 | 119 | 92.4 | 88.2 | 74.2 |
|  | 405 | 117 | 116 | 92.1 | 88.2 | 74 |
|  | 407 | 114 | 115 | 92.1 | 87.7 | 73.9 |
| From 2^nd^ replicate | 141 | 165 | 161 | 98 | 82.9 | 64 |
|  | 139 | 163 | 162 | 97.1 | 83.5 | 64.3 |
|  | 138 | 161 | 162 | 96.9 | 82.6 | 64.5 |
|  | 137 | 160 | 159 | 96.6 | 82.3 | 64.7 |
|  | 135 | 157 | 161 | 95.5 | 82.2 | 64.9 |
|  | 133 | 154 | 159 | 95.4 | 82.3 | 65.1 |
|  | 133 | 155 | 159 | 95 | 82.1 | 65.1 |
|  | 131 | 153 | 161 | 94.7 | 82.1 | 65.4 |
|  | 131 | 151 | 157 | 94.1 | 81.7 | 65.7 |
|  | 130 | 150 | 158 | 94 | 81.8 | 65.6 |
|  | 128 | 149 | 157 | 93.4 | 81.8 | 65.6 |
|  | 128 | 150 | 157 | 93.2 | 81.6 | 65.7 |
|  | 127 | 149 | 155 | 92.7 | 81.3 | 65.8 |
|  | 126 | 146 | 154 | 92.2 | 81.1 | 65.9 |
|  | 125 | 143 | 154 | 91.6 | 81.1 | 65.9 |
|  | 124 | 145 | 154 | 91.3 | 81.1 | 65.8 |
|  | 123 | 141 | 152 | 91.1 | 80.7 | 66 |
|  | 123 | 140 | 152 | 90.9 | 80.7 | 65.9 |
|  | 122 | 143 | 153 | 90.4 | 80.5 | 66 |
|  | 119 | 139 | 152 | 90.1 | 80.1 | 66 |
|  | 118 | 136 | 152 | 89.7 | 80.1 | 65.9 |
|  | 118 | 137 | 151 | 89.5 | 80 | 66 |
|  | 117 | 136 | 150 | 89.1 | 80 | 65.9 |
|  | 117 | 133 | 150 | 88.8 | 79.5 | 66 |
|  | 117 | 133 | 150 | 88.3 | 79.2 | 66 |
|  | 116 | 132 | 149 | 88.1 | 79.2 | 66 |
|  | 115 | 132 | 150 | 87.8 | 79.6 | 65.9 |
|  | 114 | 130 | 148 | 87.5 | 79.1 | 65.9 |
|  | 113 | 129 | 148 | 87 | 78.7 | 65.7 |
|  | 113 | 126 | 147 | 87 | 78.4 | 65.5 |
| From 3^rd^ replicate | 274 | 133 | 152 | 88.1 | 14.4 | 95.5 |
|  | 271 | 131 | 149 | 87.3 | 14.3 | 96.4 |
|  | 260 | 129 | 149 | 87.1 | 14.3 | 96.9 |
|  | 256 | 126 | 148 | 86.8 | 14.3 | 97.3 |
|  | 231 | 127 | 145 | 86.1 | 14.2 | 97.5 |
|  | 224 | 126 | 144 | 85.9 | 14.2 | 97.8 |
|  | 218 | 125 | 144 | 85.2 | 14.2 | 97.8 |
|  | 216 | 124 | 141 | 85.3 | 14.1 | 97.9 |
|  | 208 | 124 | 139 | 84.5 | 14.1 | 98.1 |
|  | 209 | 122 | 137 | 84.5 | 14.1 | 98.3 |
|  | 200 | 122 | 138 | 84 | 14 | 98.5 |
|  | 193 | 124 | 143 | 84.2 | 14 | 98.8 |
|  | 191 | 121 | 153 | 83.5 | 13.9 | 98.7 |
|  | 190 | 120 | 155 | 83.2 | 13.9 | 98.9 |
|  | 188 | 119 | 157 | 82.7 | 13.9 | 99.5 |
|  | 185 | 117 | 158 | 82.6 | 13.9 | 99.1 |
|  | 182 | 118 | 162 | 82.1 | 13.8 | 99 |
|  | 177 | 117 | 165 | 82.2 | 13.8 | 99.2 |
|  | 177 | 113 | 163 | 81.6 | 13.8 | 98.9 |
|  | 167 | 112 | 169 | 81.1 | 13.7 | 99.3 |
|  | 164 | 112 | 166 | 81.1 | 13.7 | 99.5 |
|  | 161 | 112 | 168 | 80.5 | 13.7 | 99.4 |
|  | 161 | 108 | 170 | 80.6 | 13.7 | 99.6 |
|  | 158 | 108 | 171 | 80.3 | 13.7 | 99.3 |
|  | 158 | 106 | 171 | 79.9 | 13.6 | 99.6 |
|  | 154 | 106 | 172 | 79.6 | 13.6 | 99.5 |
|  | 153 | 106 | 175 | 79.4 | 13.6 | 99.8 |
|  | 151 | 102 | 172 | 78.6 | 13.6 | 99.9 |
|  | 151 | 104 | 176 | 78.9 | 13.6 | 99.4 |
|  | 147 | 104 | 217 | 78.3 | 13.6 | 99.3 |

^f^ The last 30 data points per PSA experiment were pooled by stirring condition and challenge water into sets of n=90.
